# Impulsive Decision-Making, Affective Experiences, and Parental History of Self-Injurious Thoughts and Behaviors within Parent-Adolescent Dyads

**DOI:** 10.1007/s10802-024-01194-w

**Published:** 2024-04-22

**Authors:** Ana E. Sheehan, Paula Yoela Salvador, Nadia Bounoua, Naomi Sadeh

**Affiliations:** 1 Department of Psychological and Brain Sciences, University of Delaware, 108 Wolf Hall, Newark, DE 19176 USA; 2 Department of Psychology, University of Maryland, College Park, MD USA

**Keywords:** Impulsivity, Actor partner interdependence model, Negative affect, Positive affect, Suicide

## Abstract

Impulsive decision-making, particularly during states of affective intensity, is associated with greater risk of engagement in self-injurious thoughts and behaviors (SITBs) during adolescence. The proximal (dyadic parent-adolescent affect and impulsivity) and distal (family history of SITBs) risk factors that occur within the family system could be relevant processes at stake in the intergenerational transmission of risk. The current study tests the interdependence of parent-adolescent factors associated with risk for SITBs and probes the extent to which parent-adolescent affective states influence their own (actor-effects) and each other's (partner-effects) impulsive decision-making, and further whether these relationships are moderated by a parent’s history of SITBs. Participants included 212 (106 dyads) community parents and their adolescents who completed self-report and behavioral tasks related to positive and negative affective states, impulsive decision-making, and lifetime history of SITBs. Application of the Actor-Partner Interdependence Model (APIM) revealed a partner-effect where greater parent negative affect in the past week was associated with elevated adolescent impulsive decision-making among families with a history of SITBs (Estimate = 0.66*,* Standard Error = 0.13, *p* < 0.001). In addition, a significant actor-effect was observed where greater positive affect was associated with decreased impulsive decision-making among adolescents (Estimate = -0.21*,* Standard Error = 0.10, *p* = 0.03), however, moderating effects of parent history of SITBs were not detected. Findings from the present study shed light on the interdependence of affect and impulsivity within parent-adolescent dyads, and the extent to which these interactions may be particularly salient for families with known vulnerabilities for SITBs.

## Introduction

Adolescence represents a developmental period marked by increased impulsivity and engagement in risky behaviors, including Self-Injurious Thoughts and Behaviors (SITBs), which are associated with negative mental and physical health conditions that persist into adulthood (Crone et al., 2016; Willoughby et al., 2021). One of the most widely studied constructs in relation to SITBs is impulsivity (Wenzel & Beck, 2008), a multidimensional construct, with one distinct facet involving the preference for smaller, immediate rewards over larger delayed rewards. Impulsive decision-making has been associated with more frequent engagement in impulsive behaviors including substance use, reckless driving, and self-harm (Amlung et al., 2019; Madden & Johnson, 2010; Nigg, 2017). Delay discounting paradigms have frequently been used to model this preference for impulsive decision-making and may be more sensitive to the state-dependent nature of impulsive decision-making in comparison to more traditional self-report measurements of impulsivity, which capture trait-level tendencies. Specifically, during this task participants are required to choose between receiving a smaller monetary reward now (i.e., impulsive decision-making) or a larger monetary reward later (i.e., logical choice for maximizing rewards), with varying monetary rewards (e.g., $100, $200, $300) and periods of delay (e.g., one week, one month, one year) (Koffarnus & Bickel, 2014). A systematic review of the literature by Gifuni and colleagues (2021) found that preference for impulsive decision-making on the delayed discounting task was associated with future engagement in SITBs among individuals with a history of SITBs. Further, greater difficulty delaying future rewards, or inhibiting impulsive decision-making, was found among individuals with, but not without, a family history of suicide (Bridge et al., 2015). Altogether, these findings suggest that impairments in value-based impulsive decision-making could be relevant risk factors for engagement in SITBs, particularly during adolescence.

In addition to reward dysregulation, decision-making may be further disrupted by activation of positive and negative affective states, heightening the risk for impulsive decision-making. This is particularly relevant within the context of adolescent SITBs, where emotion-based impulsive decision-making has been associated with suicide attempts (Auerbach et al., 2017; Johnson et al., 2022). However, most adolescents who experience emotion-relevant impulsive decision-making do not go on to engage in SITBs. Therefore, other factors that increase an individual’s vulnerability for future engagement in these behaviors should be considered. For example, familial genetic and nongenetic factors, including family history of SITBs, may increase the likelihood that an adolescent will engage in self-injurious behaviors within the context of emotion-relevant impulsive decision-making (Brent & Mann, 2005; Jones et al., 2021). However, to date, there remains relatively little understanding of the interdependent relationship between affective states and impulsive decision-making within parent-adolescent dyads. Therefore, research that seeks to understand mechanisms that may be at stake in the transmission of risk from parents to their adolescents warrants further exploration.

Impulsive decision-making, particularly in response to emotional states, is known to increase during adolescence (Steinberg, 2010). Negative affective states have demonstrated consistent positive associations with risk taking behaviors in youth. Prominent theoretical models of behavioral dysregulation, including the Emotional Cascade Model (Selby & Joiner, 2009), posit that, as the intensity of negative affective experiences increases, individuals may engage in high-risk behaviors (e.g., suicide and non-suicidal self-injury [NSSI]) as a means of distraction and temporary relief from emotional discomfort. Further evidence for the relationship between the trait level tendency to respond impulsively under negative affective states was associated with frequency of past month suicide attempts among a sample of adolescent inpatients (Auerbach et al., 2017). Further, a preliminary investigation of daily risk for NSSI, found that negative affective states assessed in the moment were associated with daily experiences of NSSI thoughts. These findings highlight the prominent role negative affectivity at trait and state levels may play in the perpetration of risky and impulsive decision-making (e.g., SITBs).

Alternatively, impulsive decision-making under the influence of positive emotional states may also be associated with reinforcement of risky behaviors. For example, Claes and Muehlenkamp (2013) found that increased positive urgency, or the trait level tendency to act impulsively during positive affective states, was linked to NSSI among adolescents. Furthermore, research using ecological momentary assessment (EMA) found that, among patients with bulimia nervosa, an increase in positive affect in the moment was observed following engagement in NSSI behaviors (Muehlenkamp et al., 2009). These findings are consistent with the possibility that engagement in self-injurious behaviors may serve an emotional regulatory function and the temporary increase in positive emotions immediately following engagement in NSSI could reinforce these behaviors over time.

### Adolescent-Parent Risk Transmission

Both individual-level and interpersonal processes may contribute to increased engagement in impulsive decision-making. In particular, dyadic relationships between adolescents and their parents represents a potential site of risk transmission (Brent & Mann, 2005; Ranning et al., 2022). Developmental models of emotions suggest that parents influence their children’s emotion regulation well into adolescence (Eisenberg et al., 1998). To this end, parents act as socialization agents for their children, where parental emotion dysregulation and inappropriate emotional expression, may contribute to poor emotion development outcomes for children (Bariola et al., 2011; Dix, 1991). Given that adolescence, more than other developmental stages, is marked by frequent and emotionally intense experiences, combined with the increased desire for autonomy, there are frequent opportunities for parent-child socialization processes to occur (Eisenberg & Morris, 2002; Miller-Slough & Dunsmore, 2016). Research by Maoz and colleagues (2014), for example, suggests that the offspring of parents with current mood disorders (e.g., depression) characterized by increases in negative affective states, were more likely to present with externalizing psychopathology, marked by increases in impulsivity. On the other hand, much less work has examined dyadic influences of parent positive affect on adolescent impulsivity. Greater parent positive affect has been associated with lower levels of externalizing and impulsive problems in young children (Wang et al., 2016). Furthermore, studies employing EMA to examine daily variation in parental affect found that parent variability in parent positive affect was associated with attention deficit hyperactivity disorder symptoms in youth, characterized by impulsive decision-making (Li & Lansford, 2018). Together, these findings highlight how parent affective states may be associated with impulsive action in their offspring; however, their interdependence remains poorly understood. Thus, the present study seeks to explore the interdependence of parent-adolescent affective states and impulsive decision-making processes within the transactional context of the family system.

To the extent that affect and impulsive decision-making are thought to be associated with the onset of SITBs (Auerbach et al., 2017; Lockwood et al., 2017), it would be important to evaluate how affective states and impulsive decision-making might be instantiated within the family context. Family, adoption, and twin studies have established familial transmission of self-injurious behaviors, such that rates of attempted suicide are elevated among the relatives of individuals who have died by suicide (Brent & Mann, 2005; Cheng et al., 2000; Gould et al., 1996). Moreover, it is known that family history of SITBs heightens risk for impulsive decision-making (Bridge et al., 2015), and past research has found elevated levels of impulsivity and aggression among decedents of individuals who have died by suicide (Brent et al., 2003). However, the extent to which a parent’s history of SITBs might influence bidirectional associations between parents and their adolescents' respective emotion-based impulsive decision-making has received virtually no empirical attention. Therefore, it would be important to determine first whether parents and adolescents influence each other’s affective states and impulsive decision-making, and further, whether these relationships differ based on family history of SITBs.

### Actor-Partner Models

Prior research examining parent-child relationship mechanisms of risk transmission have tended to prioritize linear models, which fail to capture the bidirectional associations between parent and their adolescents. Thus, for the present study, we implemented an actor-partner model, an approach that considers the interdependence of the family member dyads (Cook & Kenny, 2005). A major advantage of this approach lies in its ability to model both the impact parent and adolescent affect has on their own impulsive decision-making (actor effects), and the impact parent and adolescent affective states may have on each other’s impulsive decision-making (partner effects).

Past studies have applied this approach to different dyadic relationships including couples, patient-caregiver, and parent-child relationships (Driscoll et al., 2012; Israel et al., 2014; Liu et al., 2013; Maroufizadeh et al., 2018; Thomson et al., 2012). However, application of this model to understanding psychosocial factors in parent-adolescent relationships at risk for SITBs remains limited. One study examined the relationship between impulsivity and depression symptoms in an acute sample of adolescents recruited from inpatient and partial hospitalization programs and found interdependence between parent impulsivity and adolescent depression (Wolff et al., 2020). While informative, these findings may not generalize to community samples, for whom parent-adolescent interactions remain pertinent as a site of potential risk and/or protection. In addition to understanding mechanisms including impulsive decision-making and affective instability that might be at play in the transmission of risk from parents to their adolescents, it is also important to better understand potential moderators of these relationships. The current study adds to the literature by examining the role of family history of SITBs as a potential moderator in the bidirectional influences of parent-adolescent affect on impulsive decision-making. Therefore, our proposed study seeks to extend past work to address some of the previously mentioned limitations.

### Current Study

The present study examines positive and negative affective states and impulsive decision-making on a delayed discounting task among a sample of parents and their adolescents recruited from the community. We used actor-partner interdependence models (APIM) to test the interdependence of parent-adolescent relationships and probe the extent to which an adolescent’s affective state might influence their own impulsive decision-making (i.e., the preference for immediate versus delayed rewards on a delay discounting task), along with that of their parent and vice versa. Furthermore, as an exploratory aim, we examined how parents’ own history of engagement in SITBs might moderate these actor or partner relationships. To this end, our study extends upon previous work by testing the interdependence of self-reported (affect) and behavioral measures (impulsive decision-making) within parent-adolescent dyads.

We hypothesized that there would be a significant positive relationship between an individual’s affect and their impulsive decision-making (actor effects). Furthermore, based on findings from previous literature supporting intergenerational transmission of risk for impulsive decision-making and emotion dysregulation from parents to adolescents (Li et al., 2019; Wolff et al., 2020), we hypothesized greater parent positive and negative affect would be associated with greater delay discounting among adolescents (partner effect). However, less remains known about adolescent’s impact on their caregivers' impulsive decision-making. Therefore, as an exploratory aim, we also hypothesized that greater adolescent positive and negative affect would be associated with greater delay discounting among parents. Although exploratory, we hypothesized that these relationships would be moderated by parent history of SITBs, such that actor and partner effects between affect and impulsive decision-making would be strongest among dyads with parents who have a history of SITBs, based on previous literature on the intergenerational transmission of risk.

## Methods

### Participants

A sample of 106 parent-adolescent dyads (N = 212) were recruited to participate in a study examining dyadic relationships between parents and their adolescents. To increase the diversity within our sample, we posted flyers online and across a wide range of communities, including neighborhoods in Wilmington, Delaware with elevated rates of socioeconomic disadvantage and adversity (https://www.neighborhoodscout.com/de/wilmington/crime; Wilmington, DE Crime Rates, 2021). Eligible dyads were fluent in English with adolescents between the ages of 13 and 17 years old. Dyads were asked to complete a set of self-report questionnaires and delay discounting task virtually via a secure, HIPAA compliant Zoom meeting. Parents and their children logged on using separate devices (e.g., smartphone, laptop) and were consented/assented before completing the surveys.

Parents in this sample were between the ages of 32 and 62 years old (*M* = 44.33, SD = 6.63) the majority of whom were mothers (84.9%). The majority of parents identified as White (65.7%), followed by Black (28.7%), Asian (3.7%), and American Indian or Alaska Native (2.8%), with 3.8% identifying as Hispanic. The socioeconomic status of this sample was diverse, as most participants reported a yearly income between $50,000 and $100,000 (33%), followed by $50,000 or less (28.3%). Adolescents in this sample were between the ages of 13 and 17 years old (*M* = 15.02, *SD* = 1.4). The majority of adolescents identified as female (54.7%), followed by male (41.5%), and 3.8% chose Other. Adolescents identified as primarily White (65.4%), followed by Black (31.8%), Hispanic (8.7%), Asian (2.8%), American Indian or Alaska Native (1.9%), and Native Hawaiian or Other Pacific Islander (0.9%).

### Procedures

The study procedures were approved by the Institutional Review Board of Human Studies Research at the University of Delaware. The authors assert that all procedures contributing to this work comply with the ethical standards of the relevant national and institutional committees on human experimentation and with the Helsinki Declaration of 1975. Prior to data collection, informed consent and assent was obtained after reviewing the study description and procedures, and participants were compensated financially for their participation. Study data were collected, managed, and stored at the University of Delaware using REDCap electronic data capture tools (Research Electronic Data Capture; Harris et al., 2019).

### Measures

#### Positive and Negative Affect Schedule (PANAS)

Positive and negative affective states were measured using the PANAS (Watson & Clark, 1994; Watson et al., 1988), a self-report questionnaire comprised of 20-items. Parents and adolescents were each asked to report to what degree they were experiencing positive and negative emotions in the past week. Sample negative affect items include: “guilty” and “hostile”, while sample positive affect items include: “enthusiastic” and “excited”. Participants were asked to report the degree to which they were feeling positive and negative emotions on a Likert scale ranging from 1 (*Very slightly or not at all*) to 5 (*Extremely*). The alphas for the subscales were good, with Cronbach’s α ranging from 0.83 to 0.90. Scoring higher on the positive and negative affect subscales indicates more positive and negative affect, respectively.

#### Delay Discounting

Both parents and adolescents were presented separately with a computer-administered task where they chose between different hypothetical monetary rewards in a 5-item questionnaire (Koffarnus & Bickel, 2014). In each scenario, participants are asked to choose between two options of receiving a small amount of money that is available to them immediately or a larger amount of money that would be awarded at a later time (e.g., Would you prefer receiving $500 now or $1,000 a year from now?). After each choice, the amount of time delayed is adjusted to determine the discount rate and time to completion for a given amount of money. Since the discount rates are not normally distributed, the estimated *k*-values were natural-log-transformed. The larger the ln *k* value, the greater the preference for the smaller immediate monetary choice (greater impulsive decision-making) compared to the larger delayed monetary choice.

#### Self-Injurious Thoughts and Behaviors (SITBs)

The Risky, Impulsive, and Self-destructive Behavior Questionnaire (RISQ; Sadeh & Baskin-Sommers, 2017) is a 38-item self-report questionnaire that was used to assess various risky and impulsive behaviors among adults across the lifespan. SITBs were assessed with four items on the RISQ asking participants how many times they had *“Thought about killing yourself”, “Tried to kill yourself”, “Cut, burned, or hurt yourself on purpose without trying to die”, or “Had a plan to kill yourself”* across their lifespan. A dichotomous SITB variable was created with parents who endorsed any of the following lifetime SITB items receiving a score of 1 and all others receiving a score of 0.

#### Data Analysis

Preliminary analyses including descriptive statistics were conducted using IBM SPSS statistics version 28. We conducted a set of paired sample t-tests to examine whether parents and adolescents present with differing patterns for all study variables. In addition, we examined bivariate correlations between study variables.

We applied the actor-partner interdependence model, which accounts for the non-independence of parent and adolescent dyads, to examine the relationship between parent and teen negative and positive affect and its associations with parent and teen delay discounting scores. APIM analyses were conducted using a structural equation modeling approach with full information maximum likelihood estimation and 5000 bootstrap resampling procedures to generate bias-corrected confidence intervals in Mplus Version 8.1 (Muthén & Muthén, 1998). In this way, APIM models allow for the evaluation of parent and adolescent affect on their own tendencies to delay immediate reward (actor effect) and on the tendency to delay immediate reward of the other dyad member (partner effect). Here the actor effects represent estimates that control for the partner effects, with the reverse also being true (Cook & Kenny, 2005). To this end, two separate APIM models examined 1) the effects of positive affect on delay discounting and 2) the effects of negative affect on delay discounting. Models considered to fit the observed data were indicated by a nonsignificant chi‐square statistic, a root mean squared error of approximation (RMSEA) of less than or equal to 0.05, a comparative fit index (CFI), and Tucker‐Lewis index (TLI) of greater than 0.90. To examine the moderating effect of family history of SITBs, we used multi-group analysis in Mplus to examine whether the APIM models differed between families with versus without a history of SITBs. That is, we examined the moderating role of family history of SITBs on APIM models evaluating 1) the effects of positive affect on delay discounting and 2) the effects of negative affect on delay discounting. For all models, we report standardized (*β)* effect estimates. In line with effect size conventions for linear relationships (Cohen, 2013), we consider *β* = 0.10 a small effect*, β* = 0.30 a medium effect, and* β* = 0.50 a large effect.

## Results

### Preliminary Analyses

In order to examine if parents and adolescents presented differing patterns of positive and negative affect in relation to delay discounting, we conducted a set of paired sample t-tests (Table 1). Our results suggest that adolescents reported significantly greater negative affect than parents. Conversely, parents endorsed significantly greater positive affect than adolescents. There were no significant differences between parent and adolescent delay discounting scores.

**Table 1:** Comparison of parent and adolescent scores on key study variables

Variable	Parent M(SD) [range]	Adolescent M(SD) [range]	T(df)	95% CI	*p-value*	Cohen’s *d*
Negative Affect^a^	16.38 (5.75)	18.52 (6.85)	2.41(92)	0.38 – 3.90	0.02	8.56
	[10 – 33]	[9 – 40]				
Positive Affect^a^	32.71 (8.15)	28.27 (7.46)	-4.08 (92)	-6.60 – -2.28	< 0.001	10.5
	[17 – 50]	[10 – 43]				
Delay Discounting	-5.35 (1.65)	-5.37 (2.33)	0.07 (93)	-0.48 – 0.52	0.94	2.44
	[-8.18 – -0.90]	[-9.12 – 3.18]				

*CI* Confidence Interval

^a^Positive and Negative Affect measured using the Positive and Negative Affect Schedule

Bivariate correlations examining the associations between study variables are presented in Table 2. These results indicated that adolescent delay discounting was positively associated with parent negative affect and parent delay discounting, suggesting more adolescent delay discounting (greater preference for impulsive decision-making) was related to greater parent negative affect and delay discounting. Adolescent delayed discounting was also associated with both parent and adolescent SITB history. In addition, parent delay discounting was negatively associated with teen negative affect and parent positive affect, suggesting more parent delay discounting (greater preference for impulsive decision-making) is associated with reduced adolescent negative affect and reduced parent positive affect.

**Table 2:** Bivariate correlation between key study variables

	1	2	3	4	5	6	7
1. Parent NA							
2. Teen NA	0.08						
3. Parent PA	-0.02	-0.14					
4. Teen PA	-0.03	-0.19	0.10				
5. Parent DD	-0.02	-0.26*	-0.20*	-0.14			
6. Teen DD	0.21*	0.15	-0.05	-0.06	0.29*		
7. Parent History of SITBs	0.27**	0.15	-0.17	0.05	0.08	0.28**	
8. Adolescent History of SITBs	0.25*	0.24*	-0.16	-0.10	0.04	0.26*	0.36**

*DD* Delay Discounting, *NA* Negative Affect, *PA* Positive Affect

*p<0.05; **p<0.01

### Actor-Partner Interdependence Model

**Fig. 1:**
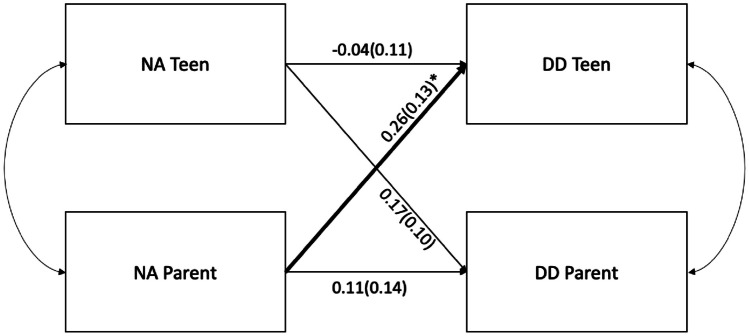
Dyadic interrelationships among adolescent and parent negative affect and delay discounting. *Note*. DD = Delay Discounting. NA = Negative Affect; coefficients are standardized with 95% confidence intervals; Bold and * denotes significant path coefficients, *p* < 0.05; model adjusted for adolescent age

**Fig. 2:**
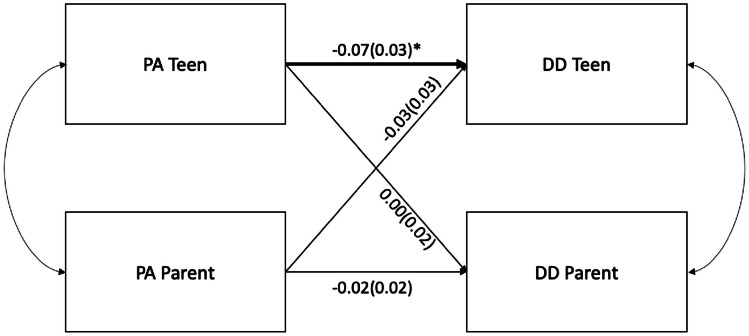
Dyadic interrelationships among adolescent and parent positive affect and delay discounting. *Note.* DD = Delay Discounting. PA = Positive Affect; coefficients are standardized with 95% confidence intervals; Bold and * denotes significant path coefficients, *p* < 0.05; model adjusted for adolescent age

A set of two actor partner interdependence structural equation modeling (SEM) models were tested (Table 3) evaluating teen and parent negative affect (Fig. 1) and positive affect (Fig. 2) as predictor variables of teen and parent delay discounting while controlling for adolescent and parent gender and age respectively.

**Table 3:** Actor Partner Interdependence Model

Effect	Role	Estimate	S.E	95% CI	p-value
**Negative Affect**					
Intercept	Adolescent	-3.51	1.00	-5.49 – -1.57	< 0.001
Actor		-0.04	0.11	-0.26 – 0.19	0.74
Partner		0.25	0.13	-0.02 – 0.49	0.04
Intercept	Parent	-3.44	1.11	-5.60 – -1.31	< 0.01
Actor		0.11	0.14	-0.16 – 0.36	0.40
Partner		0.17	0.10	-0.04 – 0.36	0.09
**Positive Affect**					
Intercept	Adolescent	-1.91	1.01	-3.93 – 0.05	0.06
Actor		-0.21	0.10	-0.38 – 0.01	0.03
Partner		-0.12	0.10	-0.31 – 0.08	0.24
Intercept	Parent	-2.27	0.97	-4.14 – -0.38	0.02
Actor		0.08	0.12	-0.29 – 0.16	0.50
Partner		0.01	0.11	-0.20 – 0.24	0.92

Results control for parent and adolescent age, which are not presented

#### Negative Affect and Delay Discounting

First, APIM evaluating interdependence between parent and adolescent negative affect and delay discounting did not detect any significant actor effects, suggesting parent and adolescent self-reported negative affect were not significantly related to their own delay discounting scores (Fig. 1). On the other hand, a significant partner effect with a small to medium effect size was observed with parent negative affect positively associated with adolescent delay discounting scores (i.e., more impulsive decision-making). With the total sample, the results explained approximately 2.8% of the variance in adolescent delay discounting and 8.8% of the variance in parent delay discounting.

#### Positive Affect and Delay Discounting

Next, APIM evaluating interdependence between parent and adolescent positive affect and delay discounting detected a significant actor effect with a small effect size, wherein adolescent positive affect was positively associated with adolescent delay discounting scores. No significant partner effects were observed, suggesting that adolescent positive affect was not associated with parent delay discounting and parent positive affect was not associated with teen delay discounting scores. With the total sample, results explained approximately 1.5% of the variance in adolescent delay discounting and 3.7% of the variance in parent delay discounting.

### History of SITBs as a Moderator of Actor Partner Interdependence Models

Next, to evaluate the impact of family history of SITBs on our models, we further tested the relationship between negative affect and delay discounting among parents with and without a history of SITBs (i.e., suicidal ideation, suicide attempt, non-suicidal self-injury, or suicidal plans). Within our sample, 28.30% of parents reported a history of SITBs. Among parents with a lifetime history of SITBs 96.67% endorsed suicidal ideation, 13.33% endorsed a history of NSSI, 16.66% endorsed a history of plans, and 33.33% endorsed a history of suicide attempts. This pattern was similar among the adolescent group, with 25.74% of the adolescents reporting a history of SITBs. Further, 57.69% of adolescents with a history of SITBs also had a parent who reported a history of SITBs.

Results from a moderated APIM model examining the relationship between delay discounting and negative affect revealed a moderating effect of parents’ history of SITBs. Specifically, our results revealed a significant partner effect with a large effect size only for the group with a family history of SITBs, where greater parent negative affect was associated with increased delay discounting values among teens (*Estimate* = *0.66,* S.E. = 0.13, *p* < *0.001;* Table 4). The APIM model among parents with a history of SITBs explained approximately 43.3% of the variance in adolescent delay discounting and 20.8% of the variance in parent delay discounting. In contrast, the APIM model among parents without a history of SITBs explained approximately 5.0% of the variance in adolescent delay discounting and 0.5% of the variance in parent delay discounting. Further, we used the model constraint function to test differences between the partner paths among groups with versus without a history of SITBs. A significant difference between the parent to adolescent partner paths was detected, wherein a stronger positive association between parent negative affect and adolescent delay discounting was observed for parents with a history of SITBs compared to the parent to adolescent partner path among parents without a history of SITBs (*Estimate* = -0.34, S.E = 0.09,* p* < 0.001). There was not a significant difference between adolescent to parent partner paths among parents with and without a history of SITBs (*Estimate* = -0.06, S.E = 0.05,* p* = 0.26).

**Table 4:** Moderated Actor Partner Interdependence Model

Effect	Role	Estimate	S.E	95% C.I.	*p*
**Negative Affect** *Parent history of SITBs*					
Intercept	Adolescent	-2.04	1.78	-5.48 – 1.46	0.25
Actor		-0.12	0.10	-0.30 – 0.09	0.24
Partner		0.66	0.13	0.34 – 0.85	< 0.001
Intercept	Parent	-2.25	1.83	-5.46 – 1.93	0.22
Actor		-0.07	0.16	-0.22 – 0.40	0.65
Partner		0.31	0.19	-0.11 – 0.63	0.09
*No parent history of SITBs*					
Intercept	Adolescent	-3.23	1.32	-5.90 – -0.79	0.01
Actor		-0.14	0.12	-0.35 – 0.10	0.24
Partner		-0.15	0.14	-0.41 – 0.12	0.26
Intercept	Parent	-3.78	1.32	-6.27 – -1.15	< 0.01
Actor		0.06	0.12	-0.22 – 0.29	0.65
Partner		0.04	0.11	-0.17 – 0.27	0.73
**Positive Affect**					
*Parent history of SITBs*					
Intercept	Adolescent	-1.18	1.80	-4.79 – 2.34	0.51
Actor		-0.15	0.11	-0.39 – 0.05	0.18
Partner		0.16	0.19	-0.29 – 0.46	0.40
Intercept	Parent	-1.14	1.80	-3.91 – 1.80	0.42
Actor		-0.05	0.14	-0.33 – 0.19	0.70
Partner		0.19	0.15	-0.11 – 0.48	0.22
*No parent history of SITBs*					
Intercept	Adolescent	-2.32	1.30	-4.91 – -0.07	0.07
Actor		-0.19	0.12	-0.39 – 0.08	0.11
Partner		-0.16	0.12	-0.38 – 0.11	0.20
Intercept	Parent	-3.18	1.26	-5.62 – -0.69	0.01
Actor		-0.06	0.13	-0.29 – 0.24	0.68
Partner		-0.05	0.16	-0.34 – 0.29	0.78

Results control for parent and adolescent age, which are not presented

*SITBs* Self-Injurious Thoughts and Behaviors

In the second moderated APIM examining the relationship between delay discounting and positive affect, no significant actor or partner associations were found among families with or without a history of SITBs (Table 4). Further, when we used the model constraint function to test differences between the partner paths among groups with versus without a history of SITBs, no significant differences were detected between the parent to adolescent partner paths (*Estimate* = -0.09, S.E = 0.08,* p* = 0.22) or the adolescent to parent partner paths (*Estimate* = -0.05, S.E = 0.05,* p* = 0.33) among parents with versus without a history of SITBs.

## Discussion

The present study examined the relationship between affective states and impulsive decision-making within the context of dyadic parent-adolescent relationships using APIM. Our findings reveal a significant partner effect where greater parent negative affect during the past week was associated with elevated adolescent delay discounting (greater preference for smaller immediate monetary incentive compared to larger delayed monetary incentive). However, expected actor effects were not detected in our sample, with an exception being the significant actor effect between greater adolescent positive affect being associated with less adolescent delay discounting. To further probe potential moderators of these relationships, we examined the impact of parent’s own history of SITBs on the dyadic interactions between affective states and delay discounting. Our results indicate that significant partner effects were only observed among parents with a history of SITBs, such that parent negative affect was positively associated with adolescent delay discounting only among parents with a SITB history. Further, with regards to the relationship between delay discounting and positive affect, no significant actor or partner effects were detected among families with or without a history of SITBs. These findings provide preliminary evidence for the interdependence of impulsive decision-making and negative affect within parent-child relationships. Given negative affect and impulsive decision-making are known to be related to risk for SITBs among adolescents, our findings implicate the parent-child relationship as a potential site of risk transmission.

First, concerning the dyadic relationship between negative affect and impulsive decision-making, our findings highlight the interdependence of these factors among parents and teens as evidenced by the observed significant partner effects. As hypothesized, greater adolescent negative affect was associated with greater parent impulsive decision-making, and greater parent negative affect was associated with greater adolescent impulsive decision-making. To better understand the role of familial risk for SITBs and the relevance of affect and impulsive decision-making as a potential mechanism of risk transmission, we sought to examine the moderating impact of parent history of SITBs, defined as parents with lifetime history of suicidal ideation, attempts, plans, or NSSI, on these dyadic relationships. Our findings suggest that among dyads with a parent history of SITBs, significant partner effects emerged wherein greater parent negative affect was associated with greater adolescent impulsive decision-making. In contrast, no significant partner/actor effects emerged among dyads without a parental history of SITBs. This finding aligns with prior work indicating that negative affective states are associated with greater impulsive decision-making, where impulsive behaviors including self-harm may be implemented as a strategy to cope with aversive affective experiences (Nock, 2009; Nock & Prinstein, 2004; Selby & Joiner, 2009). Our findings expand upon this work, shedding light on the interdependence of negative affect and impulsivity within parent-adolescent dyadic relationships.

One possible interpretation of this pattern of results is that adolescents of parents with a history of SITBs may be particularly sensitive to their parents' negative affective states and seek to escape the aversive emotional experience through impulsive decision-making. This finding is supported by a robust literature highlighting a strong association between depression, characterized by increases in negative affective states, and associated increases in risk for SITBs (Moitra et al., 2021; Ribeiro et al., 2018). Parents with a history of SITBs may be more likely to also have a history of depression, which has been linked to greater prevalence of psychopathology and impulsivity in their children (Goodman et al., 2011; Stein et al., 2014). Parents with a history of depression and/or SITBs may use, and consequently model, coping strategies that are ineffective or impulsive. As a result, adolescents may be more vulnerable to adopting these ineffective coping skills, leading to more impulsive decision-making (Wolff et al., 2020). Although the current study did not directly model the influence of ineffective coping, our preliminary findings shed light on the interdependent nature of negative affect and impulsive decision-making within the parent-adolescent dyadic relationship. Future research is needed to evaluate the extent to which this interdependence may function as a mechanism of risk for SITBs in adolescence.

Our findings also align with prior research demonstrating the familial nature of SITBs (Brent & Mann, 2005) and provide some preliminary evidence for processes that could be involved in the transmission of risk through parent negative affect on adolescent impulsive decision-making. Findings from the present work are consistent with conceptual frameworks provided by Marsha Linehan (1993) who proposed that the development of self-injurious behavior is caused by transactions between the individual's own biological vulnerability to intense emotional states and an invalidating environment over time (Linehan, 1993). Situated within this framework, our findings provide support for the possibility that dyads with parents who have a history of SITBs may possess a biological vulnerability towards impulsive decision-making that is shaped, in part, by an environment that is invalidating (Crowell et al., 2014). For example, a parent who frequently experiences negative affective states may be inadvertently reinforcing an invalidating emotional environment wherein extreme negative emotional outbursts become the primary mode of communication. In an attempt to generate responses from their caregivers, adolescents may engage in more impulsive decision-making or behaviors (e.g., self-injurious behaviors), which become reinforced by parental interactions over time. Altogether, our findings provide preliminary support for the interdependence of negative affective states and impulsive decision-making within families at risk for SITBs.

Finally, with regard to dyadic relationships between positive affect and impulsive decision-making, only a significant actor effect was observed among teens, with greater positive affect associated with less impulsive decision-making. This finding is consistent with the possibility that individuals in a positive affective state may try to maintain their positive affect and refrain from engaging in behaviors, which might culminate in disruption to this positive state through loss. Furthermore, positive affect may also be associated with better use of emotion regulation strategies, which may serve as a protective mechanism against maladaptive impulsive coping behaviors in this community sample of adolescents. To this end, it is possible that teens who endorse more positive affective states are engaging in more adaptive emotion regulation strategies, which maintain positive affect and reduce the likelihood of engagement in maladaptive risky behaviors via impulsive decision-making. The absence of a partner effect, although counter to our hypothesis, suggests that among this sample, dyadic relationships between positive affect and impulsive decision-making may be less relevant. Future research could benefit from employing real-time monitoring of positive affective states and impulsive decision-making using EMA techniques to examine these complex interactions at the daily level.

Overall, our findings shed light on the interdependent nature of affective states, particularly negative affect and performance on an impulsive decision-making task within parent-child dyads with a history of SITBs by applying APIM. Importantly, this work highlights how parents and their adolescents may influence one another’s affect and impulsive decision-making, and we speculate on the role of parent-adolescent interdependence as a mechanism underlying intergenerational risk transmission. While prior research has tended to focus on parenting factors in isolation including parent emotion regulation or quality of parent-child interactions, our findings highlight the importance of examining the interdependence of these factors as they coexist within the family structure.

### Strengths and Limitations

Findings from our study must be interpreted within the context of several limitations. First, our findings represent a cross-sectional examination of affective states, impulsive decision-making, and lifetime parental history of SITBs. As such, our results do not capture the interdependence of these factors within the family dyad as they unfold over time and cannot shed light on causal pathways of risk transmission for future SITBs among adolescents. To test this possibility, future research should consider examining how these interdependent relationships might relate to the emergence of future SITBs using intensive longitudinal methods to capture thoughts and feelings preceding SITBs in the moment. In addition, our findings drew from a relatively small community sample, which may have partially contributed to the relatively smaller variance explained by our models and limit the generalizability of our findings to community samples. The sample size of the present study also prohibited examination of differences in parent-adolescent interdependence across different types of SITBs (e.g., parents with a history of attempts versus ideation). However, given that research examining parent-child dyads is relatively limited, our study provides preliminary evidence for the interdependence of parent-adolescent impulsive decision-making and negative affect. In addition, our study relied on the use of self-report questionnaires for our variables of interest with a relatively small number of items, which increases the possibility that response bias and poor reliability have influenced the findings. Future research should consider examining our variables of interest at multiple levels of analysis integrating behavioral tasks and psychophysiological data. Finally, our study enrollment was limited to one parent per adolescent, who predominantly identified as female mothers (84.9%). As a result, our findings may not be generalizable to male-identifying parents of adolescents, nor do these findings speak to how differences in family structures (e.g., single parent, stepparents) might influence our results. It would be important for future research to investigate the family system more comprehensively to better understand how dynamic processes unfold between adolescents and their caregivers. 

Despite these limitations, there are several strengths of the present study that warrant mention. First, as previously stated, these findings offer preliminary support for the interdependence of well-established risk factors for SITBs within the parent-adolescent relationship. Given that relatively little research has applied APIM to model the interdependence within parent-adolescent relationships, our findings offer an important advancement toward understanding how risk might be instantiated within the family system. Finally, a unique advantage of the present study was the examination of the moderating role of family history of SITBs, which has not been applied to work using APIM approaches to the authors’ knowledge.

Importantly, this work advances prior work investigating the interdependence of affect and impulsive decision-making within parent-adolescent dyads and the extent to which these interactions may be particularly salient for families with known vulnerabilities for SITBs. Continued examination and exploration of dyadic relationships within the family system will be an important consideration for future research aiming to uncover potential mechanisms at stake in the intergenerational transmission of risk. Further replication and extension of the present work is imperative to identify specific targets of intervention within the family system (e.g., regulation of affect and impulsive decision-making) to potentially disrupt transmission of risk for SITBs among youth.

## Data Availability

Data can be made available by contacting the corresponding author with a reasonable request.
